# RNA-Sequencing Analysis of HepG2 Cells Treated with Atorvastatin

**DOI:** 10.1371/journal.pone.0105836

**Published:** 2014-08-25

**Authors:** Camilla Stormo, Marianne K. Kringen, Robert Lyle, Ole Kristoffer Olstad, Daniel Sachse, Jens P. Berg, Armin P. Piehler

**Affiliations:** 1 Department of Medical Biochemistry, Oslo University Hospital, Ullevål, Oslo, Norway; 2 Department of Pharmacology, Oslo University Hospital, Ullevål, Oslo, Norway; 3 Department of Medical Genetics, Oslo University Hospital and University of Oslo, Oslo, Norway; 4 Institute of Clinical Medicine, Faculty of Medicine, University of Oslo, Oslo, Norway; 5 Fürst Medical Laboratory, Oslo, Norway; Queen's University Belfast, United Kingdom

## Abstract

The cholesterol-lowering drug atorvastatin is among the most prescribed drug in the world. Alternative splicing in a number of genes has been reported to be associated with variable statin response. RNA-seq has proven to be a powerful technique for genome-wide splice variant analysis. In the present study, we sought to investigate atorvastatin responsive splice variants in HepG2 cells using RNA-seq analysis to identify novel candidate genes implicated in cholesterol homeostasis and in the statin response. HepG2 cells were treated with 10 µM atorvastatin for 24 hours. RNA-seq and exon array analyses were performed. The validation of selected genes was performed using Taqman gene expression assays. RNA-seq analysis identified 121 genes and 98 specific splice variants, of which four were minor splice variants to be differentially expressed, 11 were genes with potential changes in their splicing patterns (*SYCP3, ZNF195, ZNF674, MYD88, WHSC1, KIF16B, ZNF92, AGER, FCHO1, SLC6A12* and *AKAP9*), and one was a gene (*RAP1GAP*) with differential promoter usage. The *IL21R* transcript was detected to be differentially expressed via RNA-seq and RT-qPCR, but not in the exon array. In conclusion, several novel candidate genes that are affected by atorvastatin treatment were identified in this study. Further studies are needed to determine the biological significance of the atorvastatin responsive splice variants that have been uniquely identified using RNA-seq.

## Introduction

Atorvastatin is an efficient competitive inhibitor of 3-hydroxy-3-methylglutaryl-Coenzyme A (HMG-CoA) reductase (HMGCR), the rate-limiting enzyme in cholesterol synthesis. Atorvastatin belongs to the statin class of drugs and is widely used to reduce cholesterol levels and the risk of cardiovascular disease. The cholesterol-lowering effect of statins is well documented. Statins block HMGCR and prevent the conversion of HMG-CoA to mevalonate and thereby decrease the level of sterol and non-sterol products derived from mevalonate, including cholesterol. As a compensatory effect, sterol-regulated genes, such as *HMGCR* and low-density lipoprotein (LDL) receptor (*LDLR*), are upregulated to increase *de novo* cholesterol synthesis and the receptor-mediated uptake of LDL-cholesterol from the blood.

Statins are generally well tolerated in most people [1]–[3]; however, there is a large amount of variability in the responses across individuals, which can be partly explained by genetic factors [4]. Interestingly, the expression level of a minor splice variant of *HMGCR* lacking exon 13 has been shown to be associated with variations in the plasma LDL-cholesterol levels and statin response [5]. Statins may also induce a range of adverse, muscle-related events in 1–5% of patients [6]. In addition, beneficial effects of statins on endothelial function, inflammation, and plaque stability have been demonstrated, suggesting that statins have effects beyond lowering cholesterol [7]. The exact molecular mechanisms underlying these LDL-cholesterol independent effects of statins are still unclear.

Studies on global gene expression effects of statin treatment have mostly been performed by microarray analysis [8]–[13]. Microarrays have been used in gene expression studies for nearly two decades, whereas RNA sequencing (RNA-seq) is a relatively new approach for this purpose [14], [15]. Briefly, RNA is converted into cDNA, which is fragmented and PCR-amplified before being subjected to parallel sequencing to generate millions of reads. The gene expression and splice variant levels are determined using the RNA-seq data by counting the number of sequence reads aligned to each gene in the genome.

Human hepatoma HepG2 cells are considered a useful model for studying the effects of statin treatment on hepatocytes [16]–[18]. Because alternative splicing has been reported to be relevant for cholesterol homeostasis and variation in statin response in a number of genes [5], [19]–[23], we sought to investigate statin responsive splice variant expression changes to identify novel candidate genes as potential regulators of the statin response. In this study, we used RNA-seq in combination with microarray analysis to provide a comprehensive transcriptome profile of HepG2 cells exposed to atorvastatin.

## Methods

### Cell culture and treatment

The human hepatoblastoma cell line HepG2 (American Type Culture Collection, Manassas, VA, USA) was maintained in collagen I-coated tissue culture flasks (BD Biosciences, San Jose, CA, USA) and modified Eagle's minimal essential medium (MEM; ATCC), supplemented with 10% heat inactivated fetal bovine serum (FBS) and 1% penicillin-streptomycin-glutamine mixture (Sigma-Aldrich, St Louis, MO, USA). In the treatment experiments, cells were seeded at 2×10^5^ cells/mL in a 12-well collagen I-coated plate (BD Biosciences). The following day, the medium was changed to 1 mL growth medium containing 3 mg/mL of human lipoprotein deficient serum (LPDS; Millipore, Billerica, MA, USA) instead of FBS. Because we wanted to study the effects of statin that were due to the inhibition of cholesterol biosynthesis, we used the magnitude of increased *HMGCR* mRNA expression as a marker for statin response. The initial experiments showed that HepG2 cells grown in medium containing 10% FBS had low *HMGCR* mRNA levels, which remained unchanged when treated with atorvastatin. We therefore took advantage of LPDS to activate cholesterol biosynthesis and increase the expression of *HMGCR* in both the control and atorvastatin-treated cells, as previously demonstrated [24]. In the dose response experiments, cells were treated with water dissolved (3S, 5S)-atorvastatin sodium salt (Toronto Research Chemicals North York, Ontario, Canada) at various concentrations (0, 2.5, 5, 10, 20 and 40 µM) for 24 hours in four independent experiments. For RNA-seq and exon array experiments, cells were treated with or without 10 µM atorvastatin for 24 hours in triplicate wells.

### RNA isolation

The cells were lysed by the addition of 700 µL QIAzol Lysis Reagent. The phase separation was achieved by applying 2 mL of Phase Lock Gel Heavy (5PRIME Inc., Gaithersburg, MD, USA) to the lysates. RNA was then purified from the aqueous phase using the miRNeasy Mini Kit (Qiagen, Venlo, The Netherlands), according to the manufacturer's instructions. The RNA was eluted in 30 µL of RNase/DNase-free water and stored at −80°C until analysis. The A260/A280 ratio and RNA concentration were determined using a NanoDrop ND-1000 spectrophotometer (NanoDrop Technologies, Wilmington, DE, USA). The RNA quality was assessed by microfluidic capillary electrophoresis using an Agilent 2100 Bioanalyzer and the RNA 6000 Nano Chip kit (Agilent Technologies, Santa Clara, CA, USA). An A260/A280 ratio in the range of 1.8 to 2.0 and an RIN>7 were considered acceptable. RNA samples were denatured for 2 min at 70°C prior to cDNA synthesis.

### RNA-seq

Total RNA (500 ng) from each sample was prepared using TruSeq RNA sample prep reagents (Illumina, San Diego, CA, USA) according to manufacturer's instructions, with fragmentation for 4 min at 94°C. The amplified fragmented cDNA of ∼200 bp in size were sequenced in paired-end mode using the HiSeq 2000 (Illumina) with a read length of 2×100 bp. Two FASTQ files were generated for each sample. The alignment of the reads onto the UCSC reference genome (hg19) and splice site identification were performed using Bowtie/TopHat, with mapping allowing up to two mismatches [25]. An average of 25.8±0.34 (mean ± STD) and 24.0±0.42 million 2×100-bp reads per control and atorvastatin-treated sample were obtained, respectively. The aligned reads were assembled into transcripts using the Cufflinks software [26]. Cufflinks computes normalized values termed FPKM (fragments per kilobase of exon per million fragments mapped), which reflect the mRNA expression levels [26]. The reads were mapped to a total of 23,138 Refseq genes and 39,843 Refseq transcript splice variants. Statistical analysis of differentially expressed genes and transcript splice variants, differential splicing and differential promoter usage was performed using Cuffdiff, which is integrated into Cufflinks. In the differential splicing analysis, Cuffdiff calculates the changes in the relative abundance of splice variants produced from a single primary transcript sharing a common transcription start site, such as a change in the splicing pattern. In the differential promoter usage analysis, Cuffdiff tests for differential promoter use in genes with two or more promoters that generate primary transcripts with different start sites. The status code “OK” in Cuffdiff indicates that there are a sufficient number of reads in a locus to make a reliable calculation. The default false discovery rate (FDR) of Cuffdiff is 5%. The results were visualized using the CummeRbund package [27] in the statistics environment R [28]. The CummeRbund package is available from the Bioconductor website [29]. Sequence reads have been deposited in the NCBI BioSample database (http://www.ncbi.nlm.nih.gov/biosample) with the following accessions: SAMN02808181, SAMN02808182, SAMN02808183, SAMN02808184, SAMN02808185 and SAMN02808186.

### Exon Array

Total RNA (100 ng) from each sample was prepared using the Ambion WT Expression Kit (Ambion Inc., Austin, TX, USA), according to manufacturer's instructions. Fragmented and labeled sense strand DNA was hybridized to the GeneChip Human Exon 1.0 ST Array (Affymetrix, Santa Clara, CA, USA). The array contained approximately four probes per exon and 40 probes per gene. The arrays were washed and stained using an FS-450 fluidics station (Affymetrix) and were scanned using a Hewlett Packard Gene Array Scanner 3000 7G (Hewlett Packard, Palo Alto, CA, USA). The scanned images were then analyzed using the Affymetrix GeneChip Command Console. The CEL files were imported into the Partek Genomics Suite software (Partek, Inc., St. Louis, MO) for data analysis. Robust microarray analysis (RMA) was applied for normalization. The exon array data were filtered to include only those probe sets derived from the core meta-probe list, representing approximately 17,000 RefSeq genes and full-length GenBank mRNAs. The gene expression level was estimated by averaging all of the core probe sets for that gene. Paired sample t-tests were performed to analyze the differential gene expression between the control and atorvastatin-treated HepG2 samples. A Benjamini-Hochberg correction was used to correct for multiple comparisons. A 5% FDR was considered statistically significant. The data have been deposited in NCBI's Gene Expression Omnibus [30] and are accessible through the GEO Series accession number GSE57071 (http://www.ncbi.nlm.nih.gov/geo/query/acc.cgi?acc=GSE57071).

### Comparison between RNA-seq and exon array

The strength of the linear relationship between the RNA-seq and exon array data was measured using Pearson's correlation coefficient on the log_2_ values obtained for the 17,151 genes that were detected using both methods. A constant offset of 1 was added to the FPKM values to handle the log_2_ transformation of zero values.

### Pathway analysis of differentially expressed gene lists

Statistically significant genes were imported into Ingenuity Pathway Analysis (www.ingenuity.com; Ingenuity Systems, Redwood City, CA, USA). The Core analysis was used to identify the main biological functions and canonical pathways associated with the differentially expressed genes.

### Validation by reverse transcription quantitative PCR (RTqPCR)

Total RNA (200 ng) was subjected to cDNA synthesis in a 20 µL reaction using the qScript cDNA Synthesis Kit (Quanta BioSciences, Gaithersburg, MD, USA) according to the manufacturer's instructions and stored at −20°C until analysis. All qPCRs were performed on a ViiA 7 Real-Time PCR System in a standard 96-well format in a 20 µL reaction mixture containing 10 µL of TaqMan Universal PCR Master Mix, 7 µL of RNase-free water, 1 µL of Taqman gene expression assay and 2 µL of a 1∶5 dilution of each cDNA sample (Applied Biosystems, Foster City, CA, USA). The cycling steps were 10 min at 95°C followed by 40 cycles of 95°C for 15 s and 60°C for 1 min. The following genes and Taqman gene expression assays (Applied Biosystems) were selected to validate the RNA-seq results: *HMGCR* (Hs01102990_m1), *IL21R* (Hs00222310_m1), *PPIA* (Hs99999904_m1) and *TBP* (Hs99999910_m1). *PPIA* and *TBP* have previously been validated in our laboratory to be suitable for normalization in atorvastatin-treated HepG2 cells [24]. Each sample was run in duplicate. The quantification cycle (Cq) values were normalized according to the ΔΔCq method for calculating fold changes in the mRNA levels [31]: ΔΔCq = 2^−(ΔCq_treated – ΔCq_non-treated)^

## Results

### Dose-response curve

The mRNA levels of *HMGCR* in HepG2 cells increased in response to increasing concentrations of atorvastatin (Figure S1). The increases in the HMGCR mRNA levels after 24 h of atorvastatin treatment were 1.2±0.06-fold (mean±S.E at the concentration of 2.5 µM atorvastatin), 1.2±0.16-fold (5 µM), 1.7±0.19-fold (10 µM), 2.4±0.26-fold (20 µM) and 2.6±0.29-fold (40 µM) compared to that of the untreated HepG2 cells (1.0±0.21). The concentration of 10 µM atorvastatin was within the linear range of the dose-response curve and was therefore used in subsequent experiments.

### RNA-seq

#### Expression level

**Figure 1:**
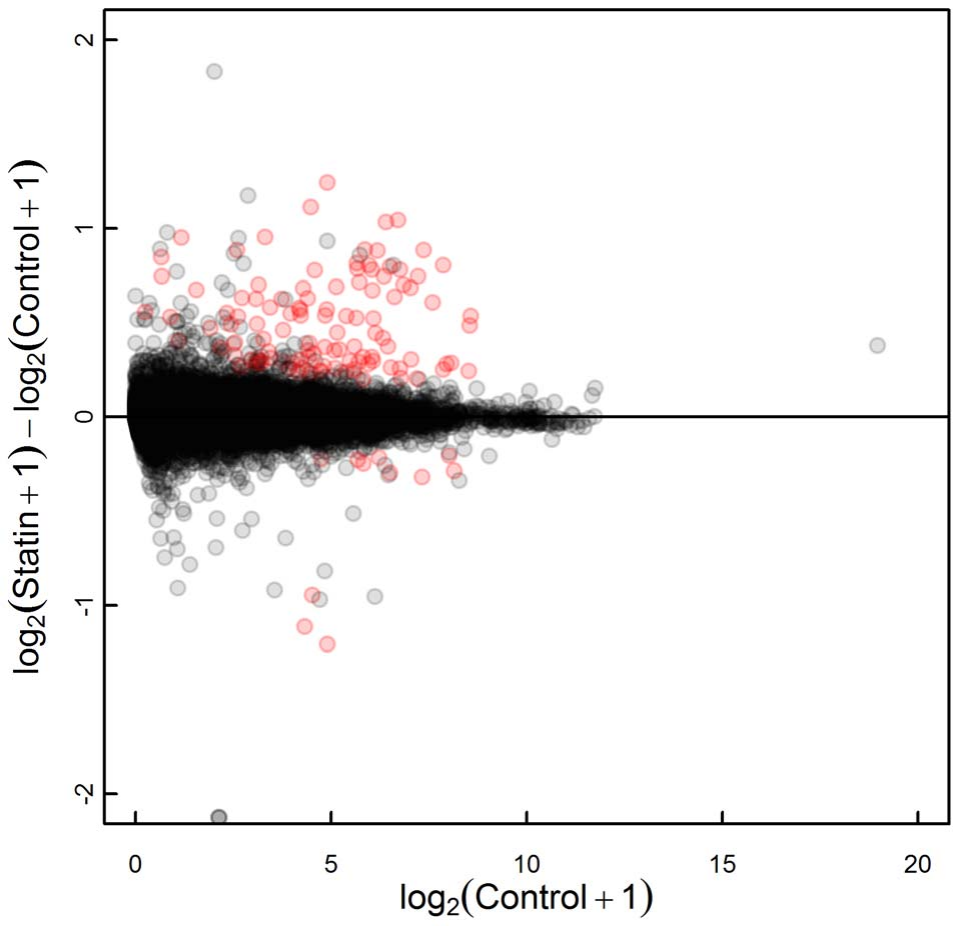
Bland-Altman plot for the comparison of FPKM expression values of the control and atorvastatin-treated HepG2 cells. Significantly differentially expressed genes are highlighted in red (n = 121).

A graph of the atorvastatin FPKM versus control FPKM expression values is shown in Figure 1. A total number of 12,426 genes were expressed when filtering for the test status "OK" in the output file containing the differential expression results. The median FPKM was 9.1 (interquartile range IQR 3.3–23) in the control samples and 9.2 (IQR 3.4–23) in the atorvastatin-treated samples.

#### Differential gene expression analysis

Cuffdiff identified 121 genes to be differentially expressed, with an expected FDR of 5%. A total of 110 genes were upregulated, and 11 genes were downregulated by atorvastatin treatment. The differentially expressed genes showed at least a 1.2-fold change in expression. The statistically significant fold changes ranged from -2.4-fold to +4.1-fold. *HMGCR* and *LDLR* expression, which can be used as cellular markers of an *in vitro* statin response, were significantly increased by 1.8-fold and 1.5-fold, respectively. The 121 differentially expressed genes were subjected to Ingenuity Pathway Analysis. The top five biological functions (Table 1 and Table S1) and canonical pathways (Table 2 and Table S2) associated with these genes are shown. Lipid metabolism and the cholesterol biosynthesis pathway were the major affected biological processes, and this finding supports the on-target effects of the *in vitro* atorvastatin treatment.

**Table 1:** Top biological functions identified by Ingenuity Pathway Analysis affected by atorvastatin treatment.

Molecular and Cellular Functions	*p*-value	# Genes
Lipid Metabolism	3.87E-24 - 1.61E-02	65
Small Molecule Biochemistry	3.87E-24 - 1.61E-02	68
Vitamin and Mineral Metabolism	3.87E-24 -1.63E-02	36
Molecular Transport	1.25E-14 - 1.63E-02	51
Nucleic Acid Metabolism	1.41E-08 - 1.61E-02	14
**Diseases and Disorders**		
Cardiovascular Disease	4.77E-11 - 1.72E-02	16
Metabolic Disease	4.77E-11 - 1.61E-02	36
Endocrine System Disorders	2.97E-09 - 1.61E-02	18
Neurological Disease	7.49E-08 - 1.61E-02	40
Psychological Disorders	7.49E-08 - 2.37E-07	20
**Physiological System Development and Function**		
Digestive System Development and Function	2.93E-06 - 2.93E-06	8
Hepatic System Development and Function	2.93E-06 - 1.61E-02	9
Organ Morphology	2.93E-06 - 1.72E-02	20
Organismal Development	6.72E-05 - 1.72E-02	20
Tissue Morphology	3.85E-04 - 1.61E-02	23

The number of differentially expressed genes associated with functional categories in atorvastatin treated HegG2 cells is shown. Note that several genes are represented in more than one category. A complete list of the biological functions and associated genes is presented in Table S1.

**Table 2:** Top five canonical pathways associated with the differentially expressed genes (n = 121) in atorvastatin treated HepG2 cells identified by Ingenuity Pathway Analysis.

Top canonical Pathways	*p*-value	Ratio
Cholesterol Biosynthesis	5.46E-25	12/13(0.92)
Cholesterol Biosynthesis II (via 24,25-dihydrolanosterol)	5.46E-25	12/13(0.92
Cholesterol Biosynthesis III (via Desmosterol)	5.46E-25	2/13 (0.92)
Zymosterol Biosynthesis	1.89E-10	5/6 (0.83)
Mevalonate Pathway I	2.17E-10	6/12 (0.5)

Canonical pathways are ordered by p-value. The ratio shows the number of genes in the dataset divided by the number of genes in the pathway. A complete list of the canonical pathways and associated genes is presented in Table S2.

#### Differential expression analysis of transcript splice variants

**Figure 2:**
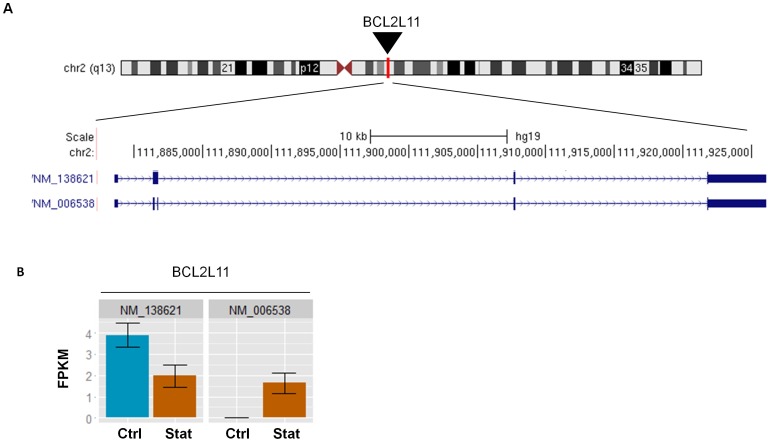
Transcript splice variants and expression of the *BCL2L11* gene. (A) The canonical splice variant with accession number NM_138621 and the alternatively spliced variant NM_006538 of *BCL2L11* are shown. Sixteen additional transcript variants of *BCL2L11* are annotated in the National Center for Biotechnology Information (NCBI) Reference Sequence (RefSeq) database (not shown here). Exons are represented by blue boxes separated by intervening sequences (introns). (B) The CummeRbund expression bar plot is shown. FPKM, fragments per kilobase of transcript per million fragments mapped, reflects the mRNA expression level of NM_138621 and NM_006538 in the un-treated (Ctrl) and atorvastatin treated (Stat) HepG2 cells. The HepG2 samples showed low expression levels of the other sixteen alternatively spliced variants (FPKM<1). An asterisk indicates the statistically significant down-regulation of NM_138621 upon atorvastatin treatment after a Benjamini-Hochberg correction (5% FDR). The alternatively spliced variant NM_006538 was slightly upregulated.

A total of 98 known transcript splice variants were differentially expressed in atorvastatin-treated samples with an FDR of 5% (Table S3). A total of 91 transcript splice variants were upregulated, and seven transcript splice variants were downregulated. The differentially expressed transcript splice variants showed at least a 1.2-fold change in expression. The statistically significant fold changes ranged from -2.3-fold to +4.1-fold. Eight of these 98 splice variants originated from the same genes, *i.e.*, two different transcript splice variants each of *ACLY*, *FDPS*, *HMGCS1* and *LSS* were significantly upregulated. The two lists of significantly differentially expressed genes (n = 121) and splice variants (n = 98) were then compared: 11 splice variants were not encoded by the 121 significant genes (Table 3). A barplot of one of these 11 genes, *BCL2L11*, is shown in Figure 2. By systematically exploring the expression level of each atorvastatin responsive splice variant, we discovered four minor splice variants encoded by the *ZNF195*, *SP1*, *DTNA* and *FAM189B* genes. *FAM189B* was significant in both the gene and the splice variant expression analysis.

**Table 3:** The 11 genes uniquely identified by differential expression analysis of transcript splice variants, but not identified by differential gene expression analysis, are shown.

Gene ID	Transcript	FPKM Atv	FPKM Ctr	Fold Change	Official full name	
ZNF195	NM_001242841	3.6	1.6	-2.3	zinc finger protein 195	Splice variant 4
MGC23284 (SNAI3-AS1)	NR_024402	1.1	2.2	2.1	SNAI3 antisense RNA 1 non-coding RNA	Splice variant 1
BCL2L11	NM_138621	3.9	2.0	-2.0	BCL2-like11 (apoptosis facilitator)	Splice variant 1
MICA	NM_000247	11.8	17.1	1.5	MHC class I polypeptide-related sequence A	Splice variant 1
TACC2	NM_206862	2.9	4.2	1.4	transforming, acidic coiled-coil containing protein 2	Splice variant 1
C10orf58 (FAM213A)	NM_032333	42.7	51.3	1.2	family with sequence similarity 213, member A	Splice variant 1
DTNA	NM_001198941	0.0	1.1	N/A	dystrobrevin, alpha	Splice variant 13
PLCXD2	NM_001185106	0.0	0.7	N/A	phosphatidylinositol-specific phospholipase C, X domain containing 2	Splice variant 1
ZNF419	NM_001098496	0.0	0.8	N/A	zinc finger protein 419	Splice variant 7
SP1	NM_003109	1.3	0.0	N/A	Sp1 transcription factor	Splice variant 2
ZNF674	NM_001146291	0.5	0.0	N/A	zinc finger protein 674	Splice variant 2

#### Differential promoter usage analysis

Statistical testing for differential promoter usage after atorvastatin treatment revealed *RAP1GAP* to be significant. A barplot of FPKM values with confidence intervals for *RAP1GAP* splice variants is shown in Figure S2.

#### Differential splicing analysis

A total of 11 genes had a different splicing pattern after atorvastatin treatment. These were *SYCP3, ZNF195, ZNF674, MYD88, WHSC1, KIF16B, ZNF92, AGER, FCHO1, SLC6A12* and *AKAP9*, ordered by increasing p-values (Figure S3-13, respectively).

### Exon array

#### Differential gene expression analysis

No genes were found to be differentially expressed after a Benjamini-Hochberg correction was applied to the data at a FDR of less than 5%.

### RNA-seq gene expression comparison with exon array

**Figure 3:**
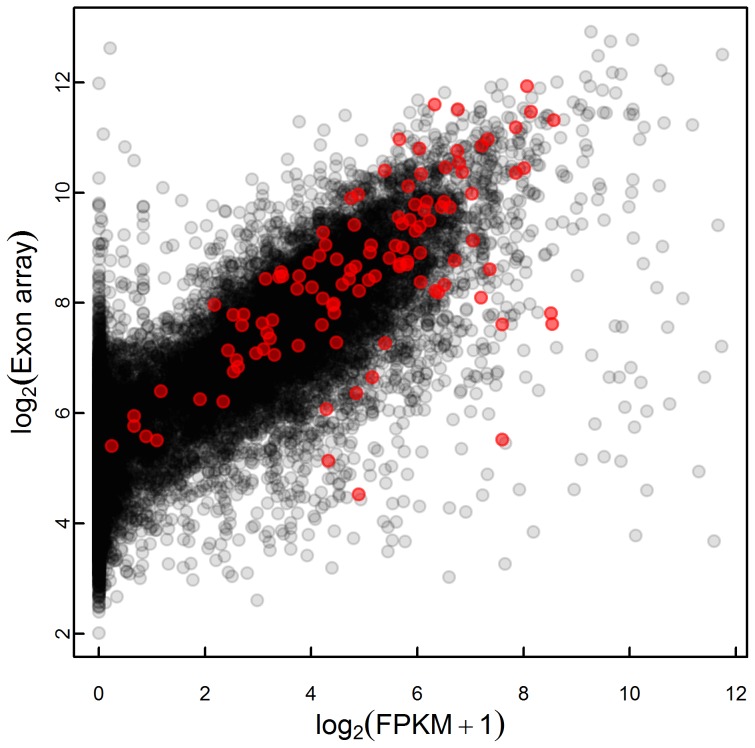
RNA-seq and exon array expression values. A comparison of RNA-seq FPKM and exon array RMA intensity values for the 17,151 genes detected using both methods in HepG2 control cells (*R* = 0.81) is shown. Significantly differentially expressed genes identified by RNA-seq are highlighted in red.

The RNA-seq and exon array data from the same samples were compared. The gene expression levels analyzed by RNA-seq and exon array were in agreement (*R* = 0.81, Figure 3). The dynamic range of RNA-seq was larger than that of the exon array; the FPKM fold changes varied from −4.3 to 4.1, whereas the exon array expression value fold changes varied from −1.6 to +1.9.

### RT-qPCR validation of differentially expressed genes

RNA-seq, exon array and RT-qPCR analysis confirmed the stable expression of *PPIA* and *TBP* between treatment groups. The fold changes of *HMGCR* mRNA levels were in good agreement among the three methods: *HMGCR* was upregulated 1.8-fold in the RNA-seq data, 1.5-fold in the exon array data and 2.2-fold in the RT-qPCR data. *IL21R* was the most upregulated gene (4.1-fold change), but its mRNA level was low (FPKM = 0.74 in atorvastatin sample). In the exon array data, *IL21R* expression was not altered by atorvastatin treatment (1.2-fold, *p* = 0.157). In the RT-qPCR data, *IL21R* was upregulated 7.1-fold. These results indicate that RNA-seq is able to quantify changes in low-abundance transcripts that were not discovered in the exon array.

## Discussion

Because the expression level of a minor transcript splice variant of *HMGCR* lacking exon 13 may explain variable cholesterol response to statins, we wanted to explore statin-induced gene expression at the level of splice variants. RNA-seq detected that the expression levels of 98 transcript splice variants were altered by atorvastatin treatment. In the majority of cases, atorvastatin changed the mRNA expression level of major splice variants. Unfortunately, there were not sufficient reads to test for the differential expression of the minor splice variant of *HMGCR* lacking exon 13. However, we discovered four minor splice variants that apparently changed more with atorvastatin than did the major ones. None of these genes were previously known to be associated with the statin response. The proteins encoded by two of the genes, *ZNF195* and *SP1*, are zinc finger transcription factors that are involved in cellular processes such as cell growth, apoptosis, differentiation and immune responses [32], [33]. The third gene, *DTNA*, encodes a protein that belongs to the dystrophin family and plays a role in synapse formation and stability, whereas the protein encoded by the fourth gene, *FAM189*, potentially binds to a WW domain-containing protein involved in apoptosis and tumor suppression [34], [35]. The significance of increased expression of these minor splice variants in statin therapy has yet to be determined.

We could confirm that statins induce the expression of a wide range of genes, most notably genes involved in lipid metabolism [13]. Furthermore, by focusing on the expression levels of splice variants, we identified 11 additional splice variants (produced from 11 different genes) that were not on the list of differentially expressed genes. For example, the canonical *BCL2L11* splice variant, which acts as an apoptotic activator, was downregulated in response to atorvastatin treatment (Figure 2), whereas the other 16 alternatively spliced variants of the *BCL2L11* gene were not affected or were only slightly upregulated. This finding shows that the regulation of single splice variants does not necessarily influence the total gene expression of a gene, suggesting that a more detailed picture is needed when investigating changes in gene expression levels.

RNA-seq technology has an advantage over microarray technology in providing information at the single-base resolution. The high resolution enables the identification of exon boundaries and thus the ability to better distinguish between different splice variants [36]. In our experiment, paired-end sequencing was performed. By using paired-end reads in the analysis, the detection of splice variants and the determination of splicing patterns are improved because cDNA fragments are sequenced from both ends [37]. It is, however, challenging that many alternative exons are expressed at rather low levels. For the majority of the genes analyzed (including *BCL2L11*), there were not sufficient reads in the locus to make a reliable calculation of differential splicing and promoter use. This may explain the minimal overlap between significant genes in the splice variant analysis (n = 98) and the differential splicing analysis (n = 11). The zinc finger transcription factor gene, *ZNF195*, was the only gene that was significant in both of the analyses.

Comparative studies of the array-based and RNA-seq platforms agree that RNA-seq is more sensitive and has a greater dynamic range than do array-based methods, with nearly no background and little technical variation [38]–[41]. The exon array data revealed that the magnitudes of expression changes were rather low after atorvastatin treatment. After correcting for multiple testing, there were no significantly differentially expressed genes in the exon array data, though there were 121 significantly differently expressed genes in the RNA-seq data. There was nevertheless a good accordance between the exon array and RNA-seq expression values, as previously demonstrated in other studies [41]. The larger dynamic range in RNA-seq data compared with the exon array allowed for the identification of the interleukin 21 receptor (*IL21R*). The differential gene expression of *IL21R* was not detected by exon array and has not previously been shown to be involved in the statin response. The upregulation of *IL21R* mRNA level was confirmed by RT-qPCR supporting the reliability of our RNA-seq data. This finding also highlights the potential of RNA-seq analysis to complement and extend microarray measurements, as noted by other researchers [15], [41]. *IL21R* encodes a receptor for interleukin 21, a group of cytokines that have immunoregulatory activity and are important in T cells, B cells, and natural killer cell responses. Statins appear to have anti-inflammatory properties independent of the lipid-lowering effect, although the clinical significance of this is unclear [42], [43]. It would be interesting to investigate the clinical role of increased *IL21R* expression in statin therapy.

We used the *HMGCR* expression levels as a marker of the statin response and found that a concentration of 10 µM gave nearly a half-maximal response in HepG2 cells cultured in LPDS medium. A high concentration of statins (100 µM) may, however, be employed when studying processes related to its toxic effects [8], [44]. For example, the number of differently expressed genes in our study using a concentration of 10 µM atorvastatin is substantially lower than that of another study, which used a 100 µM concentration of the drug (121 genes versus 1091 genes) [8]. However, in that study, the authors also identified lipid metabolism as the major biological function affected by the atorvastatin treatment, although they did not culture the cells in LPDS medium as we did. Their PCR results that demonstrated increased expression of genes involved in cholesterol metabolism (such as *ACAT2*, *HMGCS*, *HMGCR*, *SQLE*, *LSS*) were similar to our results.

In vitro effects of statins appear to be largely cell type-dependent. In epithelial cells, 10 µM atorvastatin treatments for 24 hours have been shown to induce anti-thrombotic effects due to the increased expression of genes coding for endothelial nitric oxide synthase, thrombomodulin, heat shock protein 27 and tissue plasminogen activator [45]. In the human umbilical vein endothelial cell line, EA.hy926, the increased expression of various Kruppel-like transcription factors, which have tumor-suppressing functions, and the modulation of cell cycle related genes (such as *CCNA2*, *CCNE2*, *CCNB1* and *CCNB2*) provides evidence for an anti-cancer effect of atorvastatin [46]. In human peripheral blood, an anti-inflammatory activity of atorvastatin has been detected due to decreased mRNA levels of chemokines (*CCL2*, *CCL7*, *CCL13*, *CCL18*, *CXCL1*) and cytokines (*IL-6*, *IL-8*, *IL-1*, *PAI-1*, *TGF-beta1*, *TGF-beta2*) [9]. None of these genes were significantly affected in our analysis of HepG2 cells.

A major limitation of the present study is the small number of biological replicates. Only three control samples and three atorvastatin-treated samples were analyzed. The results from our study should be replicated and presented with dose-response data to confirm the significance of the results we obtained, particularly the results from transcripts that were present at a low abundance. To conclude, we have identified several novel candidate genes that are affected by atorvastatin treatment in HepG2 cells. It would be interesting to study the biological significance of the atorvastatin-responsive splice variants that have been uniquely identified with RNA-seq.

## Supporting Information

Figure S1 Dose response curve of *HMGCR* gene expression. HepG2 cells were incubated for 24 h in culture medium containing lipoprotein deficient serum with indicated concentration of atorvastatin. *HMGCR* mRNA levels were determined by RT-qPCR using Taqman gene expression assay specific for HMGCR and two validated references genes for normalization. The results are shown as the mean fold change compared with mRNA levels in un-treated cells from four independent experiments. Error bars indicate standard errors (±S.E), n = 4.
(PNG)

Figure S2 Differential promoter usage for *RAP1GAP*. A barplot of expression values with confidence intervals for *RAP1GAP* is shown. FPKM, fragments per kilobase of transcript per million fragments mapped, reflects the mRNA expression level of transcript splice variants in the un-treated (Ctrl) and atorvastatin treated (Stat) HepG2 cells.
(JPEG)

Figure S3 Differential splicing of *SYCP3.* A barplot of expression values with confidence intervals for *SYCP3* is shown. FPKM, fragments per kilobase of transcript per million fragments mapped, reflects the mRNA expression level of transcript splice variants in the un-treated (Ctrl) and atorvastatin treated (Stat) HepG2 cells.
(PNG)

Figure S4 Differential splicing of *ZNF195*. A barplot of expression values with confidence intervals for *ZNF195* is shown. FPKM, fragments per kilobase of transcript per million fragments mapped, reflects the mRNA expression level of transcript splice variants in the un-treated (Ctrl) and atorvastatin treated (Stat) HepG2 cells.
(PNG)

Figure S5 Differential splicing of *ZNF674*. A barplot of expression values with confidence intervals for *ZNF674* is shown. FPKM, fragments per kilobase of transcript per million fragments mapped, reflects the mRNA expression level of transcript splice variants in the un-treated (Ctrl) and atorvastatin treated (Stat) HepG2 cells.
(JPEG)

Figure S6 Differential splicing of *MYD88*. A barplot of expression values with confidence intervals for *MYD88* is shown. FPKM, fragments per kilobase of transcript per million fragments mapped, reflects the mRNA expression level of transcript splice variants in the un-treated (Ctrl) and atorvastatin treated (Stat) HepG2 cells.
(PNG)

Figure S7 Differential splicing of *WHSC1*. A barplot of expression values with confidence intervals for *WHSC1* is shown. FPKM, fragments per kilobase of transcript per million fragments mapped, reflects the mRNA expression level of transcript splice variants in the un-treated (Ctrl) and atorvastatin treated (Stat) HepG2 cells.
(PNG)

Figure S8 Differential splicing of *KIF16B*. A barplot of expression values with confidence intervals for *KIF16B* is shown. FPKM, fragments per kilobase of transcript per million fragments mapped, reflects the mRNA expression level of transcript splice variants in the un-treated (Ctrl) and atorvastatin treated (Stat) HepG2 cells.
(PNG)

Figure S9 Differential splicing of *ZNF92*. A barplot of expression values with confidence intervals for *ZNF92* is shown. FPKM, fragments per kilobase of transcript per million fragments mapped, reflects the mRNA expression level of transcript splice variants in the un-treated (Ctrl) and atorvastatin treated (Stat) HepG2 cells.
(PNG)

Figure S10 Differential splicing of *AGER*. A barplot of expression values with confidence intervals for *AGER* is shown. FPKM, fragments per kilobase of transcript per million fragments mapped, reflects the mRNA expression level of transcript splice variants in the un-treated (Ctrl) and atorvastatin treated (Stat) HepG2 cells.
(PNG)

Figure S11 Differential splicing of *FCHO1*. A barplot of expression values with confidence intervals for *FCHO1* is shown. FPKM, fragments per kilobase of transcript per million fragments mapped, reflects the mRNA expression level of transcript splice variants in the un-treated (Ctrl) and atorvastatin treated (Stat) HepG2 cells.
(PNG)

Figure S12 Differential splicing of *SLC6A12*. A barplot of expression values with confidence intervals for *SLC6A12* is shown. FPKM, fragments per kilobase of transcript per million fragments mapped, reflects the mRNA expression level of transcript splice variants in the un-treated (Ctrl) and atorvastatin treated (Stat) HepG2 cells.
(PNG)

Figure S13 Differential splicing of *AKAP9*. A barplot of expression values with confidence intervals for *AKAP9* is shown. FPKM, fragments per kilobase of transcript per million fragments mapped, reflects the mRNA expression level of transcript splice variants in the un-treated (Ctrl) and atorvastatin treated (Stat) HepG2 cells.
(PNG)

Table S1 Biological functions and associated genes identified by Ingenuity Pathway Analysis in atorvastatin treated HepG2 cells.
(XLS)

Table S2 Canonical pathways and associated genes identified by Ingenuity Pathway Analysis in atorvastatin treated HepG2 cells.
(XLS)

Table S3 A comprehensive list of the 98 differently expressed splice variants identified by RNA-seq after atorvastatin treatment in HepG2 cells (cut off: l1.2l fold change, 5% FDR).
(DOC)
