# Supplementary figures and images for: RNA-Sequencing Analysis of HepG2 Cells Treated with Atorvastatin
Source: PLoS One. 2014 Aug 25;9(8):e105836. doi: 10.1371/journal.pone.0105836 (PMC4143339; doi:10.1371/journal.pone.0105836)

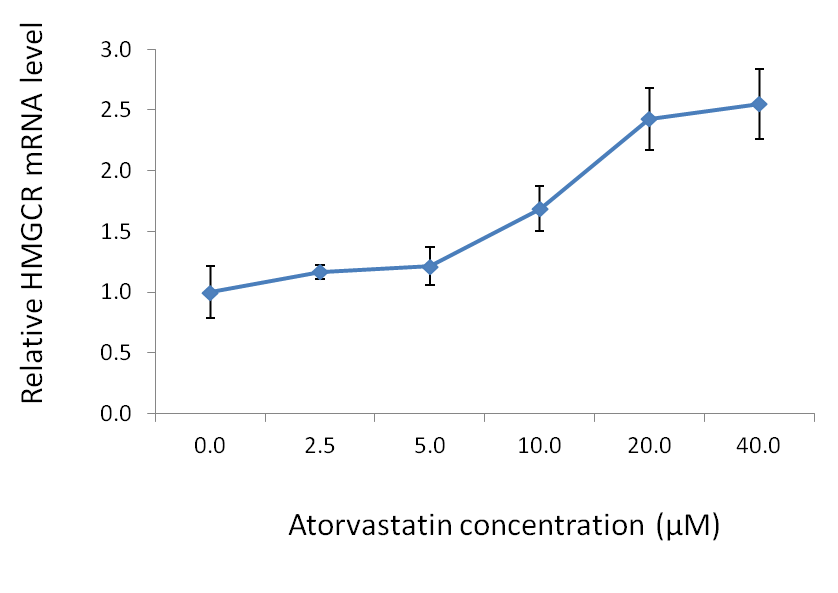

Supplement: Figure S1 — Dose response curve of HMGCR gene expression. HepG2 cells were incubated for 24 h in culture medium containing lipoprotein deficient serum with indicated concentration of atorvastatin. HMGCR mRNA levels were determined by RT-qPCR using Taqman gene expression assay specific for HMGCR and two validated references genes for normalization. The results are shown as the mean fold change compared with mRNA levels in un-treated cells from four independent experiments. Error bars indicate standard errors (±S.E), n = 4. (PNG) [file pone.0105836.s001.png]

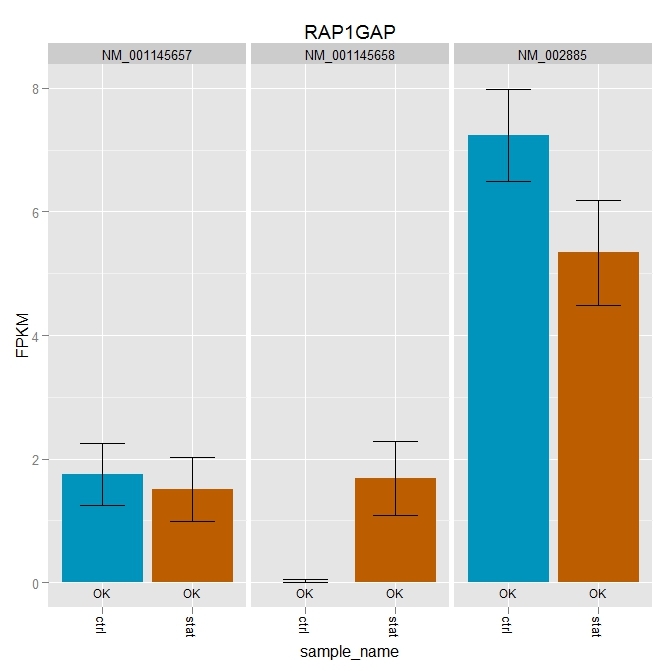

Supplement: Figure S2 — Differential promoter usage for RAP1GAP. A barplot of expression values with confidence intervals for RAP1GAP is shown. FPKM, fragments per kilobase of transcript per million fragments mapped, reflects the mRNA expression level of transcript splice variants in the un-treated (Ctrl) and atorvastatin treated (Stat) HepG2 cells. (JPEG) [file pone.0105836.s002.jpeg]

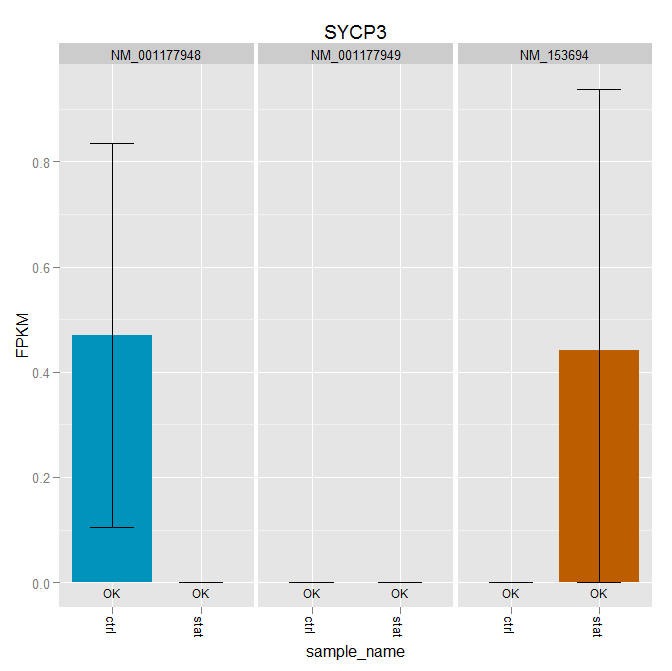

Supplement: Figure S3 — Differential splicing of SYCP3. A barplot of expression values with confidence intervals for SYCP3 is shown. FPKM, fragments per kilobase of transcript per million fragments mapped, reflects the mRNA expression level of transcript splice variants in the un-treated (Ctrl) and atorvastatin treated (Stat) HepG2 cells. (PNG) [file pone.0105836.s003.png]

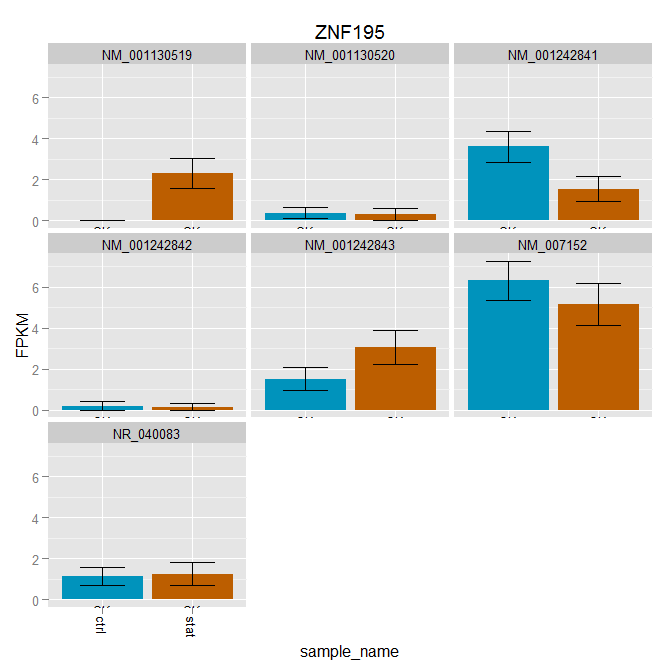

Supplement: Figure S4 — Differential splicing of ZNF195. A barplot of expression values with confidence intervals for ZNF195 is shown. FPKM, fragments per kilobase of transcript per million fragments mapped, reflects the mRNA expression level of transcript splice variants in the un-treated (Ctrl) and atorvastatin treated (Stat) HepG2 cells. (PNG) [file pone.0105836.s004.png]

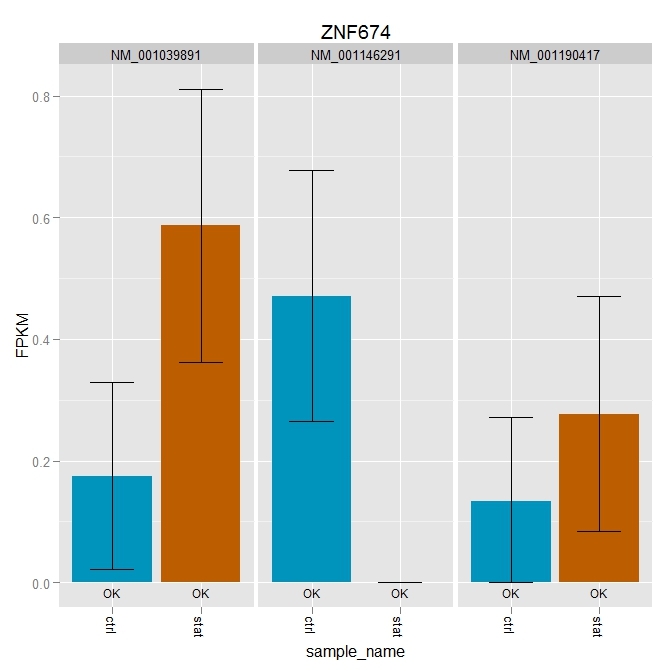

Supplement: Figure S5 — Differential splicing of ZNF674. A barplot of expression values with confidence intervals for ZNF674 is shown. FPKM, fragments per kilobase of transcript per million fragments mapped, reflects the mRNA expression level of transcript splice variants in the un-treated (Ctrl) and atorvastatin treated (Stat) HepG2 cells. (JPEG) [file pone.0105836.s005.jpeg]

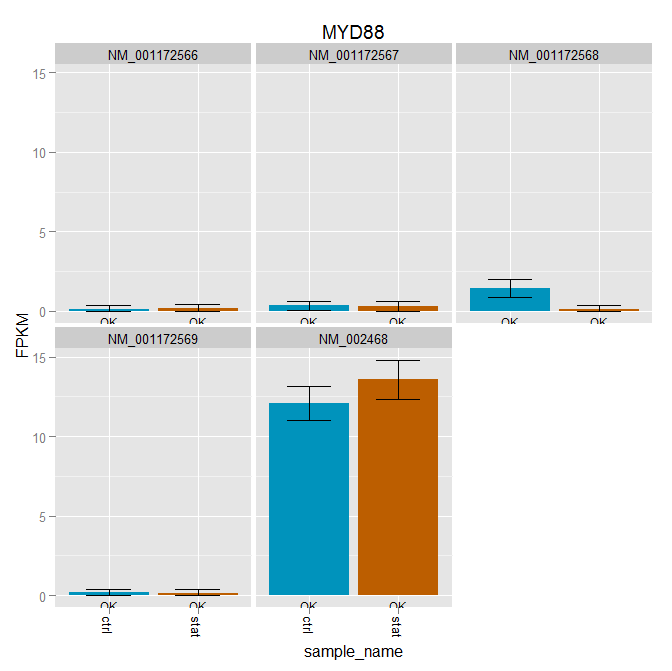

Supplement: Figure S6 — Differential splicing of MYD88. A barplot of expression values with confidence intervals for MYD88 is shown. FPKM, fragments per kilobase of transcript per million fragments mapped, reflects the mRNA expression level of transcript splice variants in the un-treated (Ctrl) and atorvastatin treated (Stat) HepG2 cells. (PNG) [file pone.0105836.s006.png]

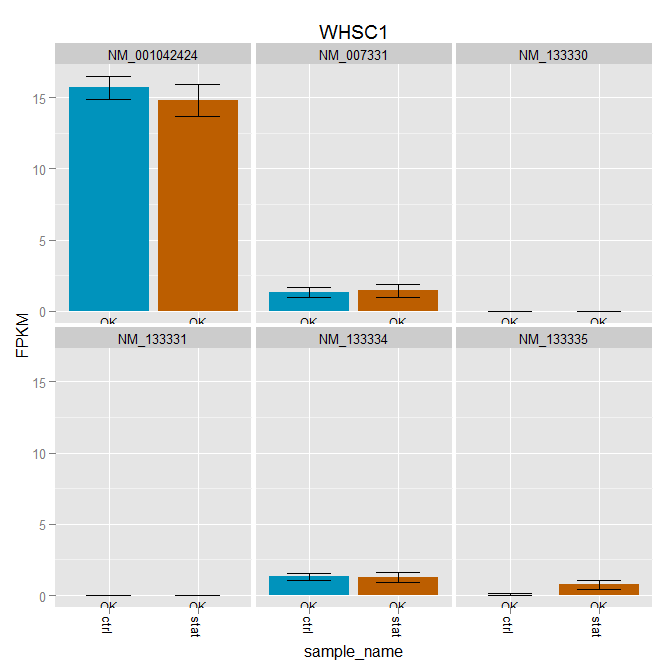

Supplement: Figure S7 — Differential splicing of WHSC1. A barplot of expression values with confidence intervals for WHSC1 is shown. FPKM, fragments per kilobase of transcript per million fragments mapped, reflects the mRNA expression level of transcript splice variants in the un-treated (Ctrl) and atorvastatin treated (Stat) HepG2 cells. (PNG) [file pone.0105836.s007.png]

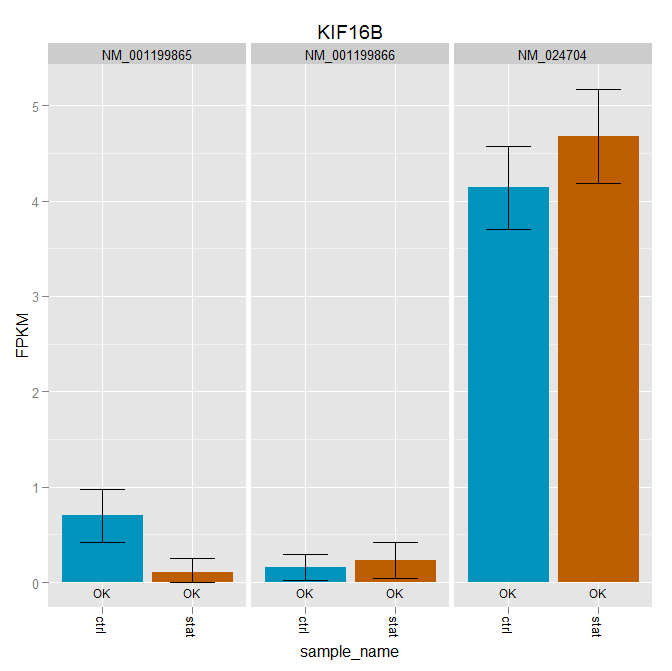

Supplement: Figure S8 — Differential splicing of KIF16B. A barplot of expression values with confidence intervals for KIF16B is shown. FPKM, fragments per kilobase of transcript per million fragments mapped, reflects the mRNA expression level of transcript splice variants in the un-treated (Ctrl) and atorvastatin treated (Stat) HepG2 cells. (PNG) [file pone.0105836.s008.png]

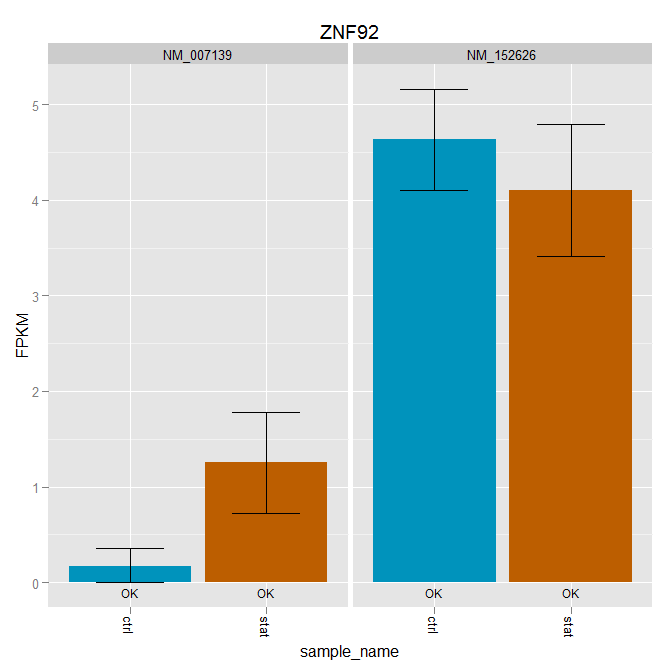

Supplement: Figure S9 — Differential splicing of ZNF92. A barplot of expression values with confidence intervals for ZNF92 is shown. FPKM, fragments per kilobase of transcript per million fragments mapped, reflects the mRNA expression level of transcript splice variants in the un-treated (Ctrl) and atorvastatin treated (Stat) HepG2 cells. (PNG) [file pone.0105836.s009.png]

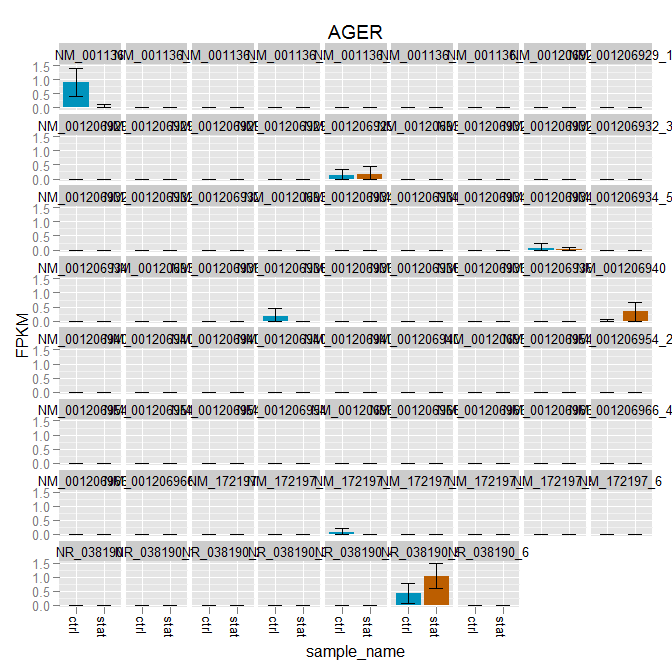

Supplement: Figure S10 — Differential splicing of AGER. A barplot of expression values with confidence intervals for AGER is shown. FPKM, fragments per kilobase of transcript per million fragments mapped, reflects the mRNA expression level of transcript splice variants in the un-treated (Ctrl) and atorvastatin treated (Stat) HepG2 cells. (PNG) [file pone.0105836.s010.png]

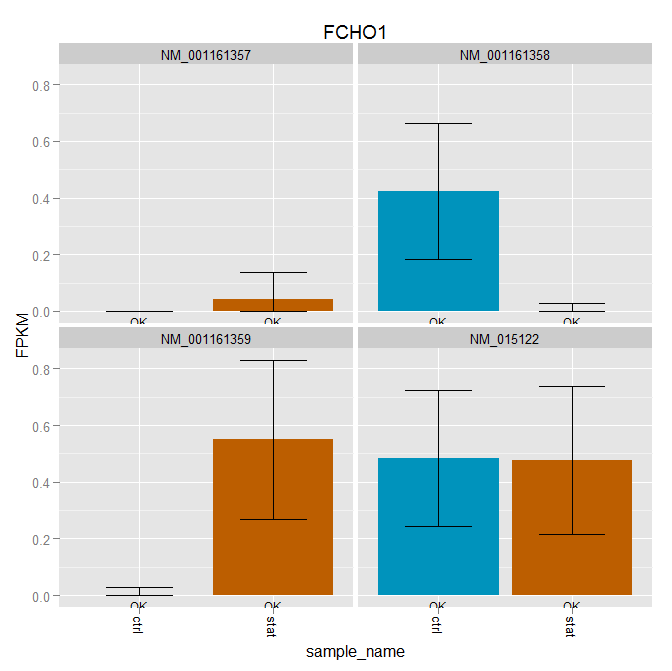

Supplement: Figure S11 — Differential splicing of FCHO1. A barplot of expression values with confidence intervals for FCHO1 is shown. FPKM, fragments per kilobase of transcript per million fragments mapped, reflects the mRNA expression level of transcript splice variants in the un-treated (Ctrl) and atorvastatin treated (Stat) HepG2 cells. (PNG) [file pone.0105836.s011.png]

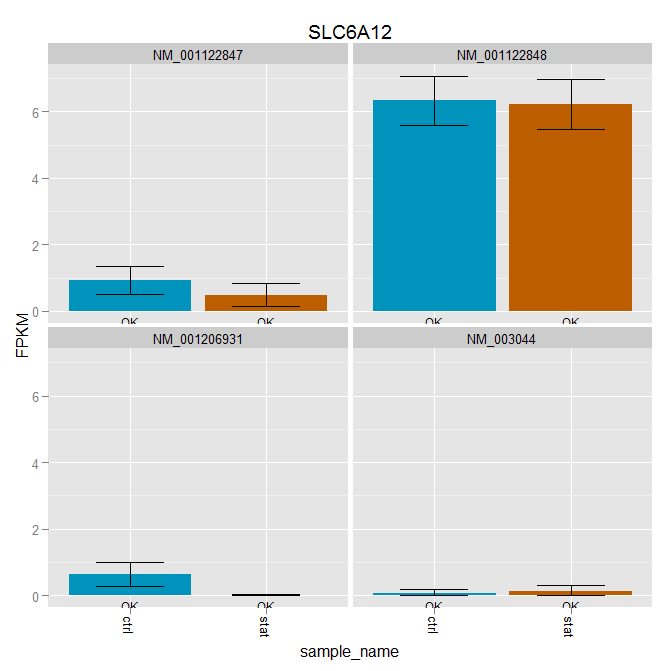

Supplement: Figure S12 — Differential splicing of SLC6A12. A barplot of expression values with confidence intervals for SLC6A12 is shown. FPKM, fragments per kilobase of transcript per million fragments mapped, reflects the mRNA expression level of transcript splice variants in the un-treated (Ctrl) and atorvastatin treated (Stat) HepG2 cells. (PNG) [file pone.0105836.s012.png]

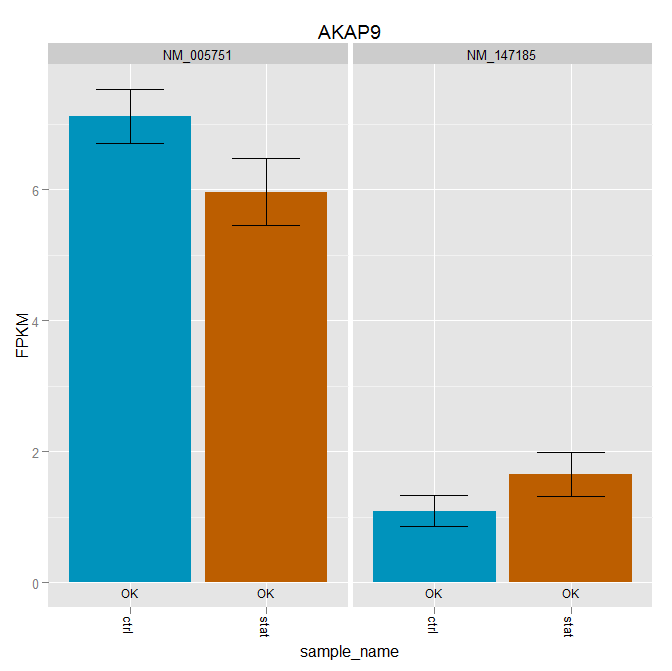

Supplement: Figure S13 — Differential splicing of AKAP9. A barplot of expression values with confidence intervals for AKAP9 is shown. FPKM, fragments per kilobase of transcript per million fragments mapped, reflects the mRNA expression level of transcript splice variants in the un-treated (Ctrl) and atorvastatin treated (Stat) HepG2 cells. (PNG) [file pone.0105836.s013.png]
