# Supplementary material for: RNA-Sequencing Analysis of HepG2 Cells Treated with Atorvastatin
Source: PLoS One. 2014 Aug 25;9(8):e105836. doi: 10.1371/journal.pone.0105836 (PMC4143339; doi:10.1371/journal.pone.0105836)
Supplement: Table S3 — A comprehensive list of the 98 differently expressed splice variants identified by RNA-seq after atorvastatin treatment in HepG2 cells (cut off: l1.2l fold change, 5% FDR). (DOC) [file pone.0105836.s016.doc]

|  | | | **Expression level (FPKM)** | | **RNA-seq** |
| --- | --- | --- | --- | --- | --- |
| **Transcript ID** | **Gene ID** |  | Ctr | Atv | Fold change |
| NM_198836 | ACACA | acetyl-CoA carboxylase alpha | 18.8 | 23.6 | 1.3 |
| NM_005891 | ACAT2 | acetyl-CoA acetyltransferase 2 | 163.8 | 303.2 | 1.9 |
| NM_001096 | ACLY | ATP citrate lyase | 71.4 | 124.3 | 1.7 |
| NM_198830 | ACLY | ATP citrate lyase | 42.3 | 61.4 | 1.5 |
| NM_001037161 | ACOT1 | acyl-CoA thioesterase 1 | 22.4 | 28.5 | 1.3 |
| NM_001995 | ACSL1 | acyl-CoA synthetase long-chain family member 1 | 20.2 | 26.1 | 1.3 |
| NM_018677 | ACSS2 | acyl-CoA synthetase short-chain family member 2 | 25.7 | 57.8 | 2.2 |
| NM_005165 | ALDOC | aldolase C, fructose-bisphosphate | 14.5 | 21.7 | 1.5 |
| NM_005498 | AP1M2 | adaptor-related protein complex 1, mu 2 subunit | 18.4 | 30.2 | 1.6 |
| NM_138621 | BCL2L11 | BCL2-like 11 (apoptosis facilitator) | 3.9 | 2.0 | -2.0 |
| NM_003670 | BHLHE40 | basic helix-loop-helix family, member e40 | 15.1 | 18.7 | 1.2 |
| NM_032333 | C10orf58 | family with sequence similarity 213, member A | 42.7 | 51.3 | 1.2 |
| NM_017622 | C17orf59 | chromosome 17 open reading frame 59 | 4.1 | 6.4 | 1.6 |
| NM_152734 | C6orf89 | chromosome 6 open reading frame 89 | 12.5 | 15.6 | 1.2 |
| NM_004344 | CETN2 | centrin, EF-hand protein, 2 | 49.0 | 71.0 | 1.5 |
| NM_001286 | CLCN6 | chloride channel, voltage-sensitive 6 | 8.5 | 12.4 | 1.5 |
| NM_001844 | COL2A1 | collagen, type II, alpha 1 | 4.9 | 7.7 | 1.6 |
| NR_002207 | CSNK2A1P | casein kinase 2, alpha 1 polypeptide pseudogene | 17.2 | 26.2 | 1.5 |
| NM_001908 | CTSB | cathepsin B | 80.5 | 65.5 | -1.2 |
| NM_000786 | CYP51A1 | cytochrome P450, family 51, subfamily A, polypeptide 1 | 66.9 | 113.4 | 1.7 |
| NM_001079862 | DBI | diazepam binding inhibitor (GABA receptor modulator) | 361.2 | 428.1 | 1.2 |
| NM_019058 | DDIT4 | DNA-damage-inducible transcript 4 | 40.9 | 48.5 | 1.2 |
| NM_014762 | DHCR24 | 24-dehydrocholesterol reductase | 265.8 | 324.5 | 1.2 |
| NM_001360 | DHCR7 | 7-dehydrocholesterol reductase | 143.7 | 242.1 | 1.7 |
| NM_001386 | DPYSL2 | dihydropyrimidinase-like 2 | 27.1 | 32.8 | 1.2 |
| NM_001198941 | DTNA | dystrobrevin, alpha | 0.0 | 1.1 | N/A |
| NM_006579 | EBP | emopamil binding protein (sterol isomerase) | 65.2 | 104.5 | 1.6 |
| NM_001398 | ECH1 | enoyl CoA hydratase 1, peroxisomal | 107.2 | 128.3 | 1.2 |
| NM_001130721 | ELOVL6 | ELOVL fatty acid elongase 6 | 6.5 | 13.2 | 2.0 |
| NM_001443 | FABP1 | fatty acid binding protein 1, liver | 370.5 | 519.5 | 1.4 |
| NM_013402 | FADS1 | fatty acid desaturase 1 | 66.2 | 95.7 | 1.4 |
| NM_004265 | FADS2 | fatty acid desaturase 2 | 71.3 | 132.2 | 1.9 |
| NM_198264 | FAM189B | family with sequence similarity 189, member B | 2.3 | 4.5 | 2.0 |
| NM_004104 | FASN | fatty acid synthase | 129.3 | 208.8 | 1.6 |
| NM_004462 | FDFT1 | farnesyl-diphosphate farnesyltransferase 1 | 230.5 | 403.4 | 1.8 |
| NM_001135822 | FDPS | farnesyl diphosphate synthase | 75.7 | 115.9 | 1.5 |
| NM_001242824 | FDPS | farnesyl diphosphate synthase | 77.3 | 124.5 | 1.6 |
| NM_005141 | FGB | fibrinogen beta chain | 73.5 | 63.3 | -1.2 |
| NM_152429 | FGFBP3 | fibroblast growth factor binding protein 3 | 2.7 | 4.2 | 1.5 |
| NM_014053 | FLVCR1 | feline leukemia virus subgroup C cellular receptor 1 | 8.0 | 10.1 | 1.3 |
| NM_005476 | GNE | Glucosamine (UDP-N-acetyl)-2-epimerase/N-acetylmannosamine kinase | 6.3 | 8.3 | 1.3 |
| NM_018645 | HES6 | hairy and enhancer of split 6 (Drosophila) | 26.7 | 39.5 | 1.5 |
| NM_000859 | HMGCR | 3-hydroxy-3-methylglutaryl-CoA reductase | 60.4 | 105.2 | 1.7 |
| NM_001098272 | HMGCS1 | 3-hydroxy-3-methylglutaryl-CoA synthase 1 (soluble) | 53.3 | 109.5 | 2.1 |
| NM_002130 | HMGCS1 | 3-hydroxy-3-methylglutaryl-CoA synthase 1 (soluble) | 49.8 | 104.1 | 2.1 |
| NM_016371 | HSD17B7 | hydroxysteroid (17-beta) dehydrogenase 7 | 20.2 | 31.8 | 1.6 |
| NR_003086 | HSD17B7P2 | hydroxysteroid (17-beta) dehydrogenase 7 pseudogene 2 | 5.6 | 9.2 | 1.6 |
| NM_005896 | IDH1 | isocitrate dehydrogenase 1 (NADP+), soluble | 230.3 | 274.3 | 1.2 |
| NM_021798 | IL21R | interleukin 21 receptor | 0.2 | 0.7 | 4.1 |
| NM_000527 | LDLR | low density lipoprotein receptor | 39.0 | 57.4 | 1.5 |
| NM_006033 | LIPG | lipase, endothelial | 0.6 | 1.7 | 2.8 |
| NM_145693 | LPIN1 | lipin 1 | 5.0 | 10.1 | 2.0 |
| NM_001145437 | LSS | lanosterol synthase (2,3-oxidosqualene-lanosterol cyclase) | 10.6 | 18.9 | 1.8 |
| NM_002340 | LSS | lanosterol synthase (2,3-oxidosqualene-lanosterol cyclase) | 37.4 | 69.1 | 1.8 |
| NM_005909 | MAP1B | microtubule-associated protein 1B | 4.8 | 6.3 | 1.3 |
| NR_024402 | MGC23284 | SNAI3 antisense RNA 1 | 1.1 | 2.2 | 2.1 |
| NM_000247 | MICA | MHC class I polypeptide-related sequence A | 11.8 | 17.1 | 1.5 |
| NM_005931_1 | MICB | MHC class I polypeptide-related sequence B | 9.5 | 12.4 | 1.3 |
| NM_052845 | MMAB | methylmalonic aciduria (cobalamin deficiency) cblB type | 9.3 | 13.6 | 1.5 |
| NM_175617 | MT1E | metallothionein 1E | 34.5 | 47.5 | 1.4 |
| NM_002461 | MVD | mevalonate (diphospho) decarboxylase | 83.2 | 171.3 | 2.1 |
| NM_000431 | MVK | mevalonate kinase | 21.9 | 39.6 | 1.8 |
| NM_000434_4 | NEU1 | sialidase 1 (lysosomal sialidase) | 86.5 | 112.3 | 1.3 |
| NM_001101648 | NPC1L1 | NPC1 (Niemann-Pick disease, type C1, gene)-like 1 | 9.9 | 12.6 | 1.3 |
| NM_015922 | NSDHL | NAD(P) dependent steroid dehydrogenase-like | 30.8 | 50.1 | 1.6 |
| NM_178129 | P2RY8 | purinergic receptor P2Y, G-protein coupled, 8 | 1.9 | 3.7 | 1.9 |
| NM_174936 | PCSK9 | proprotein convertase subtilisin/kexin type 9 | 21.2 | 47.1 | 2.2 |
| NM_002861 | PCYT2 | phosphate cytidylyltransferase 2, ethanolamine | 37.0 | 50.5 | 1.4 |
| NM_020786 | PDP2 | pyruvate dehyrogenase phosphatase catalytic subunit 2 | 5.5 | 6.8 | 1.2 |
| NM_004567 | PFKFB4 | 6-phosphofructo-2-kinase/fructose-2,6-biphosphatase 4 | 4.8 | 6.7 | 1.4 |
| NM_002631 | PGD | phosphogluconate dehydrogenase | 109.2 | 126.1 | 1.2 |
| NM_015715 | PLA2G3 | phospholipase A2, group III | 0.6 | 1.8 | 3.2 |
| NM_001185106 | PLCXD2 | phosphatidylinositol-specific phospholipase C, X domain containing 2 | 0.0 | 0.7 | N/A |
| NM_025225 | PNPLA3 | patatin-like phospholipase domain containing 3 | 12.3 | 19.6 | 1.6 |
| NM_031229 | RBCK1 | RanBP-type and C3HC4-type zinc finger containing 1 | 50.0 | 61.6 | 1.2 |
| NM_016026 | RDH11 | retinol dehydrogenase 11 (all-trans/9-cis/11-cis) | 27.6 | 36.0 | 1.3 |
| NM_152617 | RNF168 | ring finger protein 168, E3 ubiquitin protein ligase | 7.6 | 9.4 | 1.2 |
| NM_005063 | SCD | stearoyl-CoA desaturase (delta-9-desaturase) | 377.3 | 547.4 | 1.5 |
| NM_000602 | SERPINE1 | serpin peptidase inhibitor, clade E member 1 | 33.0 | 39.3 | 1.2 |
| NM_170693 | SGK2 | serum/glucocorticoid regulated kinase 2 | 7.0 | 10.7 | 1.5 |
| NM_005984 | SLC25A1 | solute carrier family 25 (mitochondrial carrier; citrate transporter), member 1 | 77.5 | 93.8 | 1.2 |
| NM_180991 | SLCO4C1 | solute carrier organic anion transporter family, member 4C1 | 1.2 | 3.3 | 2.7 |
| NM_153271 | SNX33 | sorting nexin 33 | 8.3 | 10.4 | 1.3 |
| NM_003109 | SP1 | Sp1 transcription factor | 1.3 | 0.0 | N/A |
| NM_003129 | SQLE | squalene epoxidase | 96.9 | 151.3 | 1.6 |
| NM_004599 | SREBF2 | sterol regulatory element binding transcription factor 2 | 46.9 | 56.1 | 1.2 |
| NM_139164 | STARD4 | StAR-related lipid transfer (START) domain containing 4 | 17.4 | 26.3 | 1.5 |
| NM_003167 | SULT2A1 | sulfotransferase family, cytosolic, 2A, dehydroepiandrosterone | 50.1 | 42.7 | -1.2 |
| NM_014849 | SV2A | synaptic vesicle glycoprotein 2A | 0.9 | 1.7 | 2.0 |
| NM_206862 | TACC2 | transforming, acidic coiled-coil containing protein 2 | 2.9 | 4.2 | 1.4 |
| NM_021202 | TP53INP2 | tumor protein p53 inducible nuclear protein 2 | 17.6 | 21.9 | 1.2 |
| NM_178012 | TUBB2B | tubulin, beta 2B class IIb | 35.9 | 46.3 | 1.3 |
| NM_003355 | UCP2 | uncoupling protein 2 (mitochondrial, proton carrier) | 17.6 | 26.1 | 1.5 |
| NM_012478 | WBP2 | WW domain binding protein 2 | 43.3 | 53.4 | 1.2 |
| NM_001242841 | ZNF195 | zinc finger protein 195 | 3.6 | 1.6 | -2.3 |
| NM_001098496 | ZNF419 | zinc finger protein 419 | 0.0 | 0.8 | N/A |
| NM_001146291 | ZNF674 | zinc finger protein 674 | 0.5 | 0.0 | N/A |
